# The association between migraine and Parkinson’s disease: a nationwide cohort study in Korea

**DOI:** 10.4178/epih.e2024010

**Published:** 2023-12-18

**Authors:** Woo-Seok Ha, Jaeho Kim, Hee Won Hwang, Sue Hyun Lee, Ji In Kim, Jin Yong Hong, Sang Hyun Park, Kyung Do Han, Min Seok Baek

**Affiliations:** 1 Department of Neurology, Severance Hospital, Yonsei University College of Medicine, Seoul, Korea; 2 Department of Neurology, Dongtan Sacred Heart Hospital, Hallym University College of Medicine, Hwaseong, Korea; 3 Department of Neurology, Wonju Severance Christian Hospital, Yonsei University Wonju College of Medicine, Wonju, Korea; 4 Department of Statistics and Actuarial Science, Soongsil University, Seoul, Korea; 5 Research Institute of Metabolism and Inflammation, Yonsei University Wonju College of Medicine, Wonju, Korea

**Keywords:** Migraine, Headache, Parkinson disease, Aging

## Abstract

**OBJECTIVES:**

Clinical studies have suggested an association between migraine and the occurrence of Parkinson’s disease (PD). However, it is unknown whether migraine affects PD risk. We aimed to investigate the incidence of PD in patients with migraine and to determine the risk factors affecting the association between migraine and PD incidence.

**METHODS:**

Using the Korean National Health Insurance System database (2002-2019), we enrolled all Koreans aged ≥40 years who participated in the national health screening program in 2009. International Classification of Diseases (10th revision) diagnostic codes and Rare Incurable Diseases System diagnostic codes were used to define patients with migraine (within 12 months of enrollment) and newly diagnosed PD.

**RESULTS:**

We included 214,193 patients with migraine and 5,879,711 individuals without migraine. During 9.1 years of follow-up (55,435,626 person-years), 1,973 (0.92%) and 30,664 (0.52%) individuals with and without migraine, respectively, were newly diagnosed with PD. Following covariate adjustment, patients with migraine showed a 1.35-fold higher PD risk than individuals without migraine. The incidence of PD was not significantly different between patients with migraine with aura and those without aura. In males with migraine, underlying dyslipidemia increased the risk of PD (p=0.012). In contrast, among females with migraine, younger age (<65 years) increased the risk of PD (p=0.038).

**CONCLUSIONS:**

Patients with migraine were more likely to develop PD than individuals without migraine. Preventive management of underlying comorbidities and chronic migraine may affect the incidence of PD in these patients. Future prospective randomized clinical trials are warranted to clarify this association.

## GRAPHICAL ABSTRACT

[Fig f3-epih-46-e2024010]

## Key Message

Patients with migraine are more likely to develop Parkinson’s disease than individuals without migraine. Furthermore, this association appears to be more strongly linked to the frequency of migraines (episodic vs. chronic) rather than the subtype of migraine (with aura vs. without aura).

## INTRODUCTION

Migraine is a complex brain disorder that involves vascular dysfunction, short-term nerve activation, and alterations in hormonal regulation and neuromodulation that can induce chronic physiological and anatomical changes in the brain [1-3]. Evidence shows that migraine has a high comorbidity burden with several neurological disorders, such as stroke [4], depression [5], and restless leg syndrome [6,7].

Two longitudinal studies have identified temporal associations between a prior diagnosis of migraine and an elevated risk of developing Parkinson’s disease (PD). These investigations revealed that individuals with a history of migraine exhibited a 1.6 times to 2.5 times greater risk of PD than controls [8,9]. Nonetheless, the long-term implications of migraine subtypes, the role of accompanying comorbidities, and the influence of sex on the incidence of PD in people with migraines have yet to be fully elucidated.

The incidence of PD in Korea has demonstrated a continuous upward trend, with rates climbing to 1.34 per 1,000 person-years by 2018. Over the past 15 years, the total patient count has surged, increasing by approximately 250%. Currently, the prevalence of PD among individuals aged 50 and above is estimated to be around 0.4% [10,11]. Pain, along with depression and anxiety, is common among PD patients in Korea, inflicting significant distress, diminishing patients’ quality of life, and substantially contributing to the burden for caregivers [12,13].

In this study, we investigated the incidence and risk of PD in patients with migraine using a national database in Korea. We aimed to identify various factors affecting the risk of PD to shed light on the mechanism underlying the association between migraine and PD.

## MATERIALS AND METHODS

### Data source

A retrospective, nationwide, population-based cohort study was conducted using data from the Korean National Health Insurance Service (NHIS) database. The NHIS is a mandatory insurance system in the Korea that provides medical coverage to 97% of the Korean population and Medical Aid to the remaining 3% who are at the lowest income level. As a result, the NHIS database contains information on the entire Korean population, including details on diagnostic codes, insurance types, prescriptions and demographic data, with a unique anonymous number assigned to each patient. In addition, the NHIS provides a free biennial national health screening program for all of its beneficiaries aged > 20 years. The screening program includes a medical history, laboratory tests, and anthropometric data collection.

### Study population

Among 10,628,070 Koreans who participated in the national health screening program in 2009, individuals aged ≥ 40 years were eligible for inclusion in this study. We investigated the medical records of the eligible participants obtained from the NHIS database during the period spanning from 2002 to 2019. The date of participation in the 2009 national health screening program was defined as the start date for follow-up. Patients with migraine were identified by the presence of the International Classification of Diseases, 10th revision (ICD-10) code G43 in their medical records within 12 months of study enrollment. Individuals without migraine were those who had no record of migraine from 2002 to 2009. Migraine with aura was identified using the ICD-10 code G431.

To further examine the effect of migraine chronicity, we operationally defined chronic migraine as being present in individuals who were diagnosed with migraine at least twice during an interval of > 3 months within a year, whereas the remaining individuals with migraine were considered to have episodic migraine. We excluded participants with a previous diagnosis of PD. Participants were also excluded if they were diagnosed with PD or died within 12 months of enrollment. A detailed flowchart of participant enrollment is shown in Supplementary Material 1.

### Other covariates

Individual-level covariates, such as age, sex, income level, and pre-existing comorbidities, were obtained from the NHIS database. Diagnosis of comorbidities, including hypertension, diabetes, dyslipidemia, congestive heart failure, myocardial infarction, and stroke, were based on ICD-10 codes (Supplementary Material 2). We also assessed the presence of depression and anxiety, taking into account the diagnoses made within the year preceding the enrollment date. Estimated glomerular filtration rate (eGFR) data were acquired via blood testing, and data on smoking status (never, former, or current), alcohol consumption (none, mild-to-moderate, or heavy), and physical activity level were obtained from the national health screening program survey.

Mild-to-moderate and heavy alcohol consumption were defined as the intake of < 30 g and ≥ 30 g of alcohol per day, respectively. Regular exercise was defined as performance of vigorous physical activity at least 3 times weekly or moderate or light physical activity at least 5 times weekly. This was in accordance with the American College of Sports Medicine guidelines [14].

### Outcomes

The primary endpoint of the study was the development of PD. The Korean government provides financial support to patients with rare and incurable diseases, including PD, which are specifically registered and strictly screened by the NHIS. Using the United Kingdom Parkinson’s Disease Society Brain Bank criteria, only a neurologist or neurosurgeon can register patients with PD to the Rare Incurable Diseases System (RIDS). We defined a diagnosis of PD as having a primary diagnosis of PD (ICD-10 code G20) and a new registration in the RIDS with a code for PD (V124). The date of the diagnosis was defined as the primary outcome. As a secondary outcome, we further analyzed the time from diagnosis to death for those diagnosed with PD. The detailed methodology is described in our previous study [15]. Each participant was followed up from the screening date in 2009 until December 31, 2019, unless censored due to death.

### Statistical analysis

We used a Cox proportional hazards model to estimate hazard ratios (HRs) and 95% confidence intervals (CIs) to assess and compare PD risk in individuals with and without migraine. Three progressively adjusted models were used. Model 1 involved a crude analysis without any adjustment. Model 2 was adjusted for age and sex, and model 3 was adjusted for age, sex, comorbidities (except depression and anxiety), lifestyle factors (smoking, drinking, and physical exercise statuses), eGFR, and body mass index (BMI). In sensitivity analysis, we extended the adjustments to include factors of depression and anxiety. Subgroup analyses were performed using multivariate Cox regression analysis to evaluate the effect of underlying comorbidities or demographic characteristics on PD risk in individuals with migraine. All statistical analyses were performed using SAS version 9.2 (SAS Institute Inc., Cary, NC, USA).

### Ethics statement

This study was approved by the NHIS inquiry commission and Institutional Review Board of Wonju Severance Christian Hospital (CR322303). Further, it was conducted in accordance with the principles of the Declaration of Helsinki and its later amendments. The requirement for informed consent was formally waived because of the secondary analytical study design and the use of de-identified participant data.

## RESULTS

### Population characteristics

Table 1 shows the demographic characteristics of the enrolled study population. In total, 214,193 patients with migraine and 5,879,711 individuals without migraine were included in this study. The mean± standard deviation age was 54.1± 10.4 years. Compared to individuals without migraine, there were disproportionately more females than males among the patients with migraine. Hypertension, dyslipidemia, myocardial infarction, congestive heart failure, stroke, depression, and anxiety were more common in patients with migraine than in individuals without migraine, whereas diabetes was more prevalent in individuals without migraine than in patients with migraine. The proportions of never-smokers and non-drinkers were higher among patients with migraine than in individuals without migraine, whereas the proportion of regular exercisers was higher in individuals without migraine than in patients with migraine.

### Incidence of Parkinson’s disease in patients with migraine

**Figure 1.**
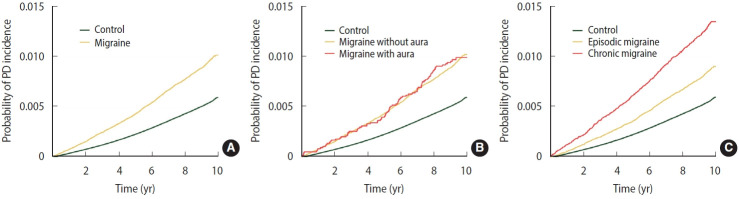
Kaplan-Meier curves of Parkinson’s disease (PD) incidence in individuals with migraine. (A) Migraine vs. no migraine. (B) Migraine with aura vs. migraine without aura vs. no migraine. (C) Chronic migraine vs. episodic migraine vs. no migraine.

Table 2 shows the Cox proportional hazard regression analysis of PD risk in patients with different types of migraine. During the 10 years of follow-up (55,435,626 person-years; median, 9.1 years), 32,637 participants (0.54%) were diagnosed with incident PD. Moreover, 1,973/214,193 (0.92%) and 30,664/5,879,711 (0.52%) individuals with and without migraine were diagnosed with PD, respectively, suggesting that patients with migraine had elevated PD risk. After adjustment for covariates, individuals with migraine showed a 1.35-fold higher risk of incident PD than those without migraine. The cumulative incidence of PD was higher in patients with migraine than in those without migraine (log-rank test, p < 0.001; Figure 1A). After adjustment for covariates, patients with migraine with and without aura (HR, 1.51; 95% CI, 1.23 to 1.86 vs. HR, 1.34; 95% CI, 1.28 to 1.40) had a higher incidence of PD than those without migraine. However, the log-rank test showed no statistically significant differences between migraineurs with and without aura (Figure 1B). Patients with episodic migraine had a 1.20-fold higher risk of developing PD than controls, while patients with chronic migraine had a 1.72-fold higher risk of developing PD. In addition, the cumulative incidence of PD was higher in patients with chronic migraine than in those with episodic migraine (log-rank test, p< 0.001; Figure 1C). In a sensitivity analysis, the risk of PD with migraine and migraine subtypes remained significant even after adjustment for depression and anxiety (HR, 1.18; 95% CI, 1.13 to 1.24 for migraine; Supplementary Material 3).

### Association between Parkinson’s disease incidence and underlying characteristics in individuals with migraine

We evaluated the risk of incident PD with stratification by age, sex, hypertension, diabetes, dyslipidemia, BMI, and current smoking status. After adjustment for covariates, the risk of PD in individuals with migraine was higher in those aged < 65 years (HR, 1.47; 95% CI, 1.36 to 1.59) than in those aged ≥ 65 years (HR, 1.28; 95% CI, 1.21 to 1.36; p= 0.006). Individuals with migraine with pre-existing dyslipidemia (HR, 1.49; 95% CI, 1.38 to 1.60) showed a higher PD risk than those with migraine who did not have dyslipidemia (HR, 1.27; 95% CI, 1.20 to 1.35; p< 0.001). Sex and underlying cardiovascular risk factors, such as hypertension, diabetes, current smoking status, and high BMI (≥ 25 kg/m^2^), did not show any interactions with the effects of migraine on PD risk (Table 3).

### Association of sex and other covariates with Parkinson’s disease in individuals with migraine

**Figure 2.**
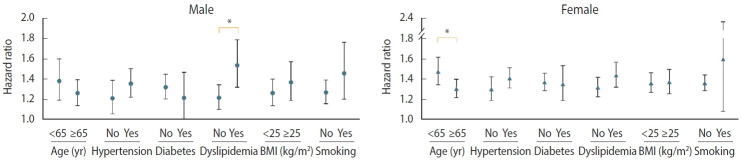
Effects of covariates on Parkinson’s disease risk in male and female with migraine. BMI, body mass index. *p<0.05.

Figure 2 shows the covariate-adjusted HRs for incident PD in each group of patients with migraine by sex. In males with migraine, the interaction between dyslipidemia and incident PD remained consistent; however, the interaction between younger age and PD risk was attenuated (HR, 1.27; 95% CI, 1.14 to 1.40 vs. HR, 1.39; 95% CI, 1.20 to 1.61; p for interaction= 0.315). In contrast, in females with migraine, the interaction between age and PD risk remained consistent; however, the interaction between dyslipidemia and PD risk was attenuated (HR, 1.44; 95% CI, 1.32 to 1.56 vs. HR, 1.32; 95% CI, 1.23 to 1.41; p for interaction= 0.125). Hypertension, diabetes, current smoking status, and high BMI (≥ 25 kg/m^2^) did not interact with the effects of migraine on PD in either sex (Supplementary Materials 4 and 5).

### Time from diagnosis of Parkinson’s disease to death in patients with migraine

In the follow-up group of patients diagnosed with PD, survival analysis showed no significant association between migraine history and time to death, even after adjusting for covariates (Table 4).

## DISCUSSION

In this large-scale, nationwide, population-based longitudinal cohort study of > 6 million individuals with a mean follow-up period of 9.1 years, we found that migraine increased the risk of incident PD. Patients with migraine with aura had a marginally higher risk of PD than those with migraine without aura, although the difference was not statistically significant. Among patients with migraine, males with a history of dyslipidemia and younger females were more likely to develop incident PD than males without dyslipidemia and older females, respectively.

To the best of our knowledge, only 2 longitudinal studies have examined the temporal relationship between migraine and PD development. One report examined interview results after 25 years of enrollment and reported that patients with migraine had a 2.3-fold to 3.6-fold higher risk of parkinsonism than individuals without migraine [8]. The increased risk of PD diagnosis was found only in patients with migraine with aura, whereas patients with migraine without aura did not have a statistically significantly increased risk of PD. The results may have been confounded by a relatively small sample size (668 patients with migraine) and underlying comorbidities in the participants, which were not accounted for in the study. In a longitudinal follow-up study using Taiwanese insurance claims data over a 31.5-month period, the HR for incident PD was 1.64-fold higher in patients with migraine than in propensity score-matched controls [9]. The potential effects related to migraine type and subgroup analyses based on underlying participant characteristics were not clear. However, the increased longitudinal risk of PD in patients with migraine is consistent with the findings of our study. Brief comparisons of the studies are shown in Supplementary Material 6.

Several mechanisms have been proposed in the aforementioned studies to explain the observed associations. Patients with migraine exhibit chronic dopaminergic hypofunction and heightened dopamine receptor sensitivity, which are closely related to the prodromal symptoms of migraine, such as yawning, nausea, and vomiting [16,17]. Consequently, it is plausible that dopaminergic dysfunction may serve as a common underlying factor in both migraine and PD, thereby explaining the positive correlation between these conditions [8,9]. Additionally, an increased accumulation of iron has been observed in individuals with migraine, which could potentially contribute to neurodegenerative diseases via oxidative stress pathways [18-20]. While we agree that these proposed mechanisms are reasonable, we posit that further mechanisms may be deduced from the results of the subgroup analyses conducted in this study.

We found that the risk of PD was higher in younger females with migraine (aged < 65 years) than in older females with migraine, although the incidence of PD has been reported to be relatively low in younger females [21]. Calcitonin gene-related peptide (CGRP) plays a significant role in the pathophysiology of migraine, such as vasodilation, neurogenic inflammation, or transmission of pain [22]. Females with migraine have higher plasma and tear CGRP levels during menstruation than females without migraine [23]. However, this difference is not observed in postmenopausal females, or in males [24]. Despite hypotheses set forth regarding the protective role of CGRP in PD pathogenesis, CGRP induces neuronal hyperexcitability and facilitates the recruitment of inflammatory mediators, which can alter the function of nicotinic receptors in the dopaminergic system in PD pathogenesis [25-27]. In addition, elevated CGRP levels have been found in the cerebrospinal fluid of patients with PD [28].

Our study additionally indicates a link between dyslipidemia and a heightened risk of PD in male with migraine. Previous studies have reported an increased incidence of dyslipidemia in migraine patients, likely due to alterations in functional arterial properties and subclinical atherosclerosis marker levels in these patients [29,30]. Dyslipidemia is also more common in PD patients than in controls [31,32]. Interestingly, the impact of cholesterol levels on PD risk seems to be sex-specific. Elevated total serum cholesterol levels have been correlated with a great risk of developing PD, particularly in males [31]. Data from the Health Professionals Follow-up Study and the Nurses’ Health Study suggest that higher intakes of animal fats and saturated fats escalate the risk of PD in men, but the same pattern was not observed in females [33]. There is also a hypothesis that α-synuclein, a protein implicated in PD, might interfere with lipid metabolism to a great extent in males. This is supported by a study in a PD mouse model showing that changes in the composition of cell membrane due to α-synuclein varied between the sexes [34].

In addition, the risk of PD was higher in patients with chronic migraine than in those with episodic migraine. We used operational definitions for both episodic and chronic migraine because it was not possible to assess migraine frequency or duration from the NHIS database. Although some portion of patients with episodic migraine may have been mixed in, we believe that most patients who actually have chronic migraine would have fallen within this operational definition of chronic migraine. The finding demonstrates the role of migraine chronicity in the development of PD. Previous magnetic resonance spectroscopic imaging studies have shown that alterations in thalamocortical pathway, which is a part of the cortico-basal ganglia circuit in PD, may contribute to migraine chronicity [35,36]. A positron-emission tomography study using (11C)PBR28, a marker of glial activation, showed that the levels of neuroinflammation in the fronto-insular cortex, basal ganglia, and primary/secondary somatosensory cortices were correlated with the frequency of migraine attacks [37]. We hypothesize that these pathophysiological changes involved in migraine chronification may increase the risk of developing PD.

This study has several limitations. First, there may have been ascertainment bias since the diagnosis of PD is made in neurology clinics. The frequent clinic visits by migraine patients could facilitate an earlier PD diagnosis in this group. However, our secondary analysis revealed that the time from PD diagnosis to death was not correlated with a history of migraine. This suggests that individuals with migraines are not merely diagnosed with PD earlier than others. Second, the diagnosis of migraine and the determination of migraine type were solely determined by ICD-10 codes applied to records within the NHIS database. In this study, the 1-year prevalence of migraine in 2009 was 3.1%. A nationwide, cross-sectional survey conducted in Korea in 2009 using the criteria of the International Classification of Headache Disorders, second edition, reported a 1-year prevalence of migraine of 6.0% [38]. Considering that only individuals who underwent medical consultations for migraine were included, we believe that the diagnosis of migraine was underestimated in this study, leading to an attenuation of our numerical results. However, the proportion of patients with migraine with aura among all patients with migraine in this study was extremely low (4.4% vs. a reported proportion of approximately 30%) [39]. This may be due to the lower prevalence of migraine with aura in Asia, a lower prevalence among those aged over 40, and the reduced validity of diagnoses based on insurance claims data compared to clinic-based or hospital-based diagnoses using structured questionnaires and clinical diagnoses. This underestimation might have contributed to the lack of significant differences in outcomes between migraineurs with and without aura. Third, some residual confounders may not have been included in the analysis. Clinical information, including prescribed medications, was not available due to the inherent limitations of the NHIS database. As described above, cholesterol-lowering medications or CGRP-modulating medications (such as triptans) might affect the development of PD. Some preventive medications for migraine, including beta-blockers, flunarizine, and valproic acid, cause rare side effects such as motor symptoms in patients with PD. Fourth, many confounders in this study (e.g., comorbidities and lifestyle factors) were assumed to be fixed, rather than time-varying, throughout the analysis [40]. Finally, this study was conducted on the Korean population, and it is unclear whether the results can be extrapolated to non-Asian populations.

Migraine is associated with the risk of developing PD. In addition, among patients with migraine, younger females (< 65 years) and males with dyslipidemia were more likely to develop PD than older females and males without dyslipidemia, respectively. We look forward to future studies to explore the underlying mechanisms and elucidate the causal relationships between migraine and PD.

## Figures and Tables

**Figure f3-epih-46-e2024010:**
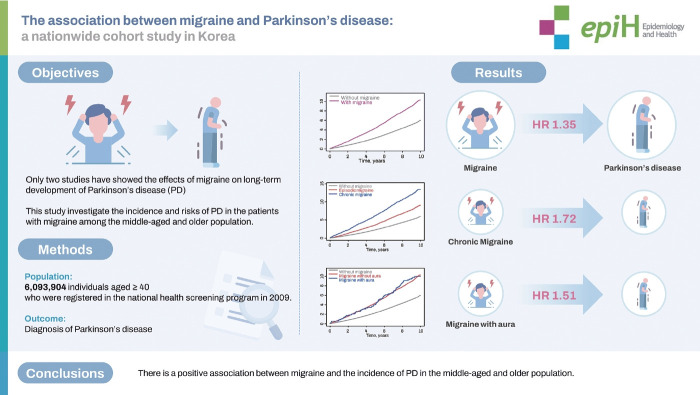


**Table 1.** Patients’ medical and demographic characteristics

Characteristics	Controls	With migraine	p-value
Total (n)	5,879,711	214,193	
Age (yr)	54.0±10.4	56.6±11.0	<0.001
Sex			
Male	3,134,537 (53.3)	59,360 (27.7)	<0.001
Female	2,745,174 (46.7)	154,833 (72.3)	
Comorbidities			
Hypertension	2,007,694 (34.2)	91,210 (42.6)	<0.001
Diabetes	684,661 (11.6)	23,978 (11.2)	<0.001
Dyslipidemia	1,244,484 (21.2)	59,652 (27.9)	<0.001
Myocardial infarction	29,304 (0.5)	1,781 (0.8)	<0.001
Congestive heart failure	42,677 (0.7)	3,313 (1.6)	<0.001
Stroke	110,832 (1.9)	12,626 (5.9)	<0.001
Depression	185,799 (3.2)	28,680 (13.4)	<0.001
Anxiety	382,769 (6.5)	57,789 (27.0)	<0.001
BMI (kg/m^2^)	23.96±3.03	24.01±3.10	<0.001
Waist circumference (cm)	81.31±8.58	80.55±8.68	<0.001
eGFR (mL/min/1.73 m^2^)	84.98±38.19	83.63±32.05	<0.001
Lowest income quartile	1,186,051 (20.2)	45,593 (21.3)	<0.001
Smoking			<0.001
Never-smoker	3,601,013 (61.2)	170,391 (79.6)	
Ex-smoker	962,970 (16.4)	20,652 (9.6)	
Current smoker	1,315,728 (22.4)	23,150 (10.8)	
Drinking			<0.001
Non-drinker	3,328,587 (56.6)	159,149 (74.3)	
Mild-to-moderate drinker	2,093,121 (35.6)	47,507 (22.2)	
Heavy drinker (≥30 g/day)	458,003 (7.8)	7,537 (3.5)	
Regular exercise	1,189,262 (20.2)	39,120 (18.3)	<0.001

Values are presented as mean±standard deviation or number (%). BMI, body mass index (calculated as weight in kilograms divided by height in meters squared); eGFR, estimated glomerular filtration rate.

**Table 2.** Cox proportional hazard regression analysis of PD risk in individuals with different migraine types^1^

Group	Participants (n)	PD diagnosis (n)	Duration (PY)	Incidence rate per 1,000 PY	HR (95% CI)		
					Model 1	Model 2	Model 3
Control	5,879,711	30,664	53,490,537	0.573	1.00 (reference)	1.00 (reference)	1.00 (reference)
Migraine	214,193	1,973	1,945,089	1.014	1.77 (1.69, 1.85)	1.40 (1.34, 1.47)	1.35 (1.29, 1.41)
Migraine without aura	204,812	1,884	1,859,445	1.013	1.77 (1.68, 1.85)	1.40 (1.33, 1.46)	1.34 (1.28, 1.40)
Migraine with aura	9,381	89	85,643	1.039	1.81 (1.47, 2.23)	1.58 (1.29, 1.95)	1.51 (1.23, 1.86)
Episodic migraine	155,469	1,253	1,413,567	0.886	1.54 (1.46, 1.63)	1.24 (1.17, 1.31)	1.20 (1.13, 1.27)
Chronic migraine	58,724	720	531,521	1.355	2.36 (2.19, 2.54)	1.81 (1.68, 1.95)	1.72 (1.60, 1.86)

PD, Parkinson’s disease; PY, person-years; HR, hazard ratio; CI, confidence interval.

1 Model 1: Unadjusted; Model 2: Adjusted for age and sex; Model 3: Adjusted for age, sex, comorbidities (hypertension, diabetes, dyslipidemia, myocardial infarction, congestive heart failure, and stroke), estimated glomerular filtration rate, body mass index, and lifestyle factors.

**Table 3.** Multivariate Cox proportional hazards regression analysis of the risk of PD in individuals with migraine

Variables		Group	Participants (n)	PD diagnosis(n)	Duration(PY)	Incidence rate per 1,000 PY	Model 3, HR (95% CI)	p-value
Age (yr)	<65	Control	4,822,074	12,696	44,537,639	0.285	1.00 (reference)	0.006
		Migraine	158,340	681	1,470,063	0.463	1.47 (1.36, 1.59)	
	≥65	Control	1,057,637	17,968	8,952,897	2.007	1.00 (reference)	
		Migraine	55,853	1,292	475,025	2.720	1.28 (1.21, 1.36)	
Sex	Male	Control	3,834,546	19,256	34,794,112	0.553	1.00 (reference)	0.336
		Migraine	138,175	1,200	1,251,030	0.959	1.32 (1.25, 1.40)	
	Female	Control	2,045,165	11,408	18,696,424	0.610	1.00 (reference)	
		Migraine	76,018	773	694,058	1.114	1.38 (1.29, 1.49)	
Hypertension	No	Control	3,872,017	14,399	35,575,317	0.405	1.00 (reference)	0.082
		Migraine	122,983	731	1,132,175	0.646	1.28 (1.19, 1.38)	
	Yes	Control	2,007,694	16,265	17,915,220	0.908	1.00 (reference)	
		Migraine	91,210	1,242	812,913	1.528	1.39 (1.31, 1.47)	
Diabetes	No	Control	5,195,050	24,481	47,478,454	0.516	1.00 (reference)	0.796
		Migraine	190,215	1,589	1,736,433	0.915	1.35 (1.29, 1.42)	
	Yes	Control	684,661	6,183	6,12,82	1.028	1.00 (reference)	
		Migraine	23,978	384	208,655	1.840	1.33 (1.20, 1.48)	
Dyslipidemia	No	Control	4,635,227	22,000	42,200,853	0.521	1.00 (reference)	<0.001
		Migraine	154,541	1,203	1,405,793	0.856	1.27 (1.20, 1.35)	
	Yes	Control	1,244,484	8,664	11,289,684	0.767	1.00 (reference)	
		Migraine	59,652	770	539,295	1.428	1.49 (1.38, 1.60)	
BMI (kg/m^2^)	<25	Control	3,134,537	15,815	28,237,213	0.560	1.00 (reference)	0.529
		Migraine	59,360	571	525,406	1.087	1.32 (1.21, 1.43)	
	≥25	Control	2,745,174	14,849	25,253,323	0.588	1.00 (reference)	
		Migraine	154,833	1,402	1,419,683	0.988	1.36 (1.29, 1.44)	
Current smoker	No	Control	4,563,983	26,984	41,645,664	0.648	1.00 (reference)	0.177
		Migraine	191,043	1,837	1,739,575	1.056	1.34 (1.27, 1.40)	
	Yes	Control	1,315,728	3,680	11,844,872	0.311	1.00 (reference)	
		Migraine	23,150	136	205,513	0.662	1.51 (1.27, 1.79)	

PD, Parkinson’s disease; PY, person-years; HR, hazard ratio; CI, confidence interval; BMI, body mass index.

**Table 4.** Cox proportional hazard regression analysis of death in patients with Parkinson’s disease^1^

Group	Participants (n)	Mortality rate (per 1,000)	HR (95% CI)			
			Model 1	Model 2	Model 3	Model 4
Control	30,664	27.7	1.00 (reference)	1.00 (reference)	1.00 (reference)	1.00 (reference)
Migraine	1,973	26.4	0.95 (0.86, 1.04)	0.98 (0.89, 1.08)	0.93 (0.84, 1.02)	0.91 (0.83, 1.01)

HR, hazard ratios; CI, confidence interval.

1 Model 1: Unadjusted; Model 2: Adjusted for age and sex; Model 3: Model 2+adjusted for comorbidities (hypertension, diabetes, dyslipidemia, myocardial infarction, congestive heart failure, and stroke), estimated glomerular filtration rate, body mass index, and lifestyle factors; Model 4: Model 3+adjusted for depression and anxiety.

## Data Availability

The data generated by this study are available from the corresponding author upon reasonable request. The data are not publicly available due to privacy restrictions.

## Supplementary materials

Supplementary Material 1. Flowchart of participant enrollment.

Supplementary Material 2. ICD-10 codes for comorbidities

Supplementary Material 3. Cox proportional hazard regression analysis of Parkinson’s disease risk in individuals with different migraine types

Supplementary Material 4. Multivariate Cox proportional hazards regression analysis for Parkinson’s disease risk in males with migraine

Supplementary Material 5. Multivariate Cox proportional hazards regression analysis for Parkinson’s disease risk in females with migraine

Supplementary Material 6. Methods, demographics, and results of the studies investigating Parkinson’s disease risk in patients with migraine
