# Supplementary material for: The association between migraine and Parkinson’s disease: a nationwide cohort study in Korea
Source: Epidemiol Health. 2023 Dec 18;46:e2024010. doi: 10.4178/epih.e2024010 (PMC10928470; doi:10.4178/epih.e2024010)
Supplement: Supplementary Material 1. — Flowchart of participant enrollment. [file epih-46-e2024010-Supplementary-1.pdf]

The association between migraine and Parkinson's disease: a nationwide cohort study

Online-Only Supplements

List of supplements

Supplementary Material 1. Flowchart of participant enrollment.

Supplementary Material 2. ICD-10 codes for comorbidities

Supplementary Material 3. Cox proportional hazard regression analysis of Parkinson's disease risk in individuals with different migraine types

Supplementary Material 4. Multivariate Cox proportional hazards regression analysis for Parkinson's disease risk in males with migraine

Supplementary Material 5. Multivariate Cox proportional hazards regression analysis for Parkinson's disease risk in females with migraine

Supplementary Material 6. Methods, demographics, and results of the studies investigating Parkinson's disease risk in patients with migraine

Supplementary Material 1. Flowchart of participant enrollment.

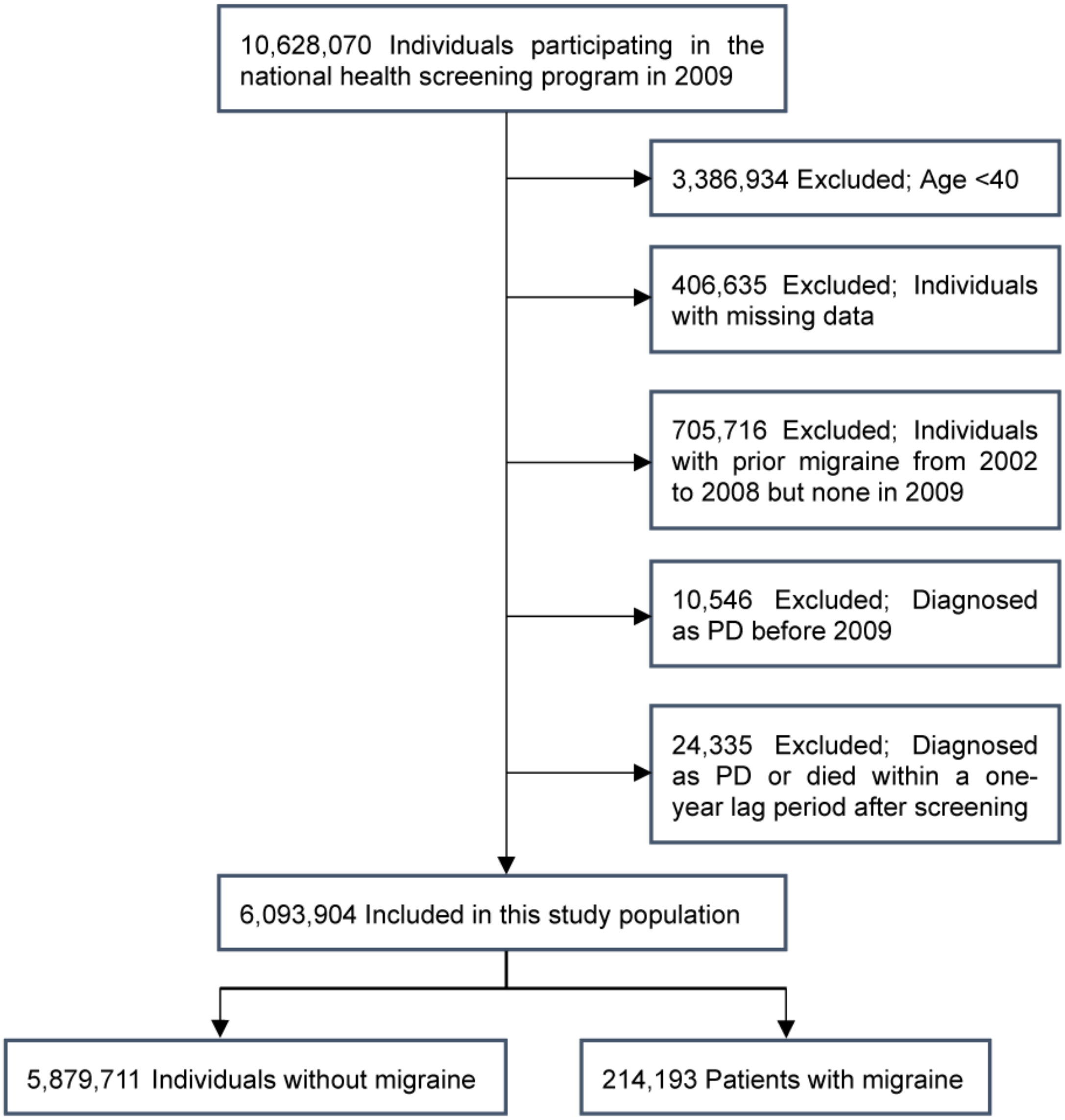

Abbreviation: PD, Parkinson's disease
