# Supplementary material for: The association between migraine and Parkinson’s disease: a nationwide cohort study in Korea
Source: Epidemiol Health. 2023 Dec 18;46:e2024010. doi: 10.4178/epih.e2024010 (PMC10928470; doi:10.4178/epih.e2024010)
Supplement: Supplementary Material 2. — ICD-10 codes for comorbidities [file epih-46-e2024010-Supplementary-2.pdf]

Supplementary Material 2. ICD-10 codes for comorbidities

Comorbidities	ICD-10 codes
**Hypertension**	I10 (essential hypertension), I13 (hypertensive heart and renal disease), or I15 (secondary hypertension)
**Diabetes**	E11 (non-insulin-dependent diabetes mellitus), E12 (malnutrition-related diabetes mellitus), E13 (other specified diabetes mellitus), or E14 (unspecified diabetes mellitus)
**Dyslipidemia**	E78 (disorders of lipoprotein metabolism and other lipidemias)
**Myocardial infarction**	I21 (ST elevation and non-ST elevation myocardial infarction), I22 (subsequent ST elevation and non-ST elevation myocardial infarction), I23 (current complications following ST elevation and non-ST elevation myocardial infarction), I24 (other acute ischemic heart diseases), or I25 (chronic ischemic heart disease)
**Congestive heart failure**	I50.0 (congestive heart failure)
**Stroke**	I63 (cerebral infarction) or I64 (stroke, not specified as hemorrhage or infarction)
**Depression**	F32 (depressive episode) or F33 (recurrent depressive disorder)
**Anxiety**	F40 (phobic anxiety disorders) or F41 (other anxiety disorders)

Abbreviation: ICD-10, International Classification of Diseases, Tenth Revision
