# Supplementary material for: The association between migraine and Parkinson’s disease: a nationwide cohort study in Korea
Source: Epidemiol Health. 2023 Dec 18;46:e2024010. doi: 10.4178/epih.e2024010 (PMC10928470; doi:10.4178/epih.e2024010)
Supplement: Supplementary Material 3. — Cox proportional hazard regression analysis of Parkinson’s disease risk in individuals with different migraine types [file epih-46-e2024010-Supplementary-3.pdf]

Supplementary Material 3. Cox proportional hazard regression analysis of Parkinson's disease risk in individuals with different migraine types

Group	Participants (n)	PD diagnosis (n)	HR (95% CI) Model 4[1]
Control	5,879,711	30,664	1 (Ref.)
Migraine	214,193	1,973	1.18 (1.13-1.24)
Migraine without aura	204,812	1,884	1.18 (1.12-1.24)
Migraine with aura	9,381	89	1.29 (1.05-1.59)
Chronic migraine	155,469	1,253	1.45 (1.35-1.57)
Episodic migraine	58,724	720	1.08 (1.02-1.14)

[1]Adjusted for age, sex, hypertension, diabetes, dyslipidemia, myocardial infarction, congestive heart failure, stroke, depression, anxiety, estimated glomerular filtration rate, body mass index, and lifestyle factors.
