# Supplementary material for: The association between migraine and Parkinson’s disease: a nationwide cohort study in Korea
Source: Epidemiol Health. 2023 Dec 18;46:e2024010. doi: 10.4178/epih.e2024010 (PMC10928470; doi:10.4178/epih.e2024010)
Supplement: Supplementary Material 4. — Multivariate Cox proportional hazards regression analysis for Parkinson’s disease risk in males with migraine [file epih-46-e2024010-Supplementary-4.pdf]

Supplementary Material 4. Multivariate Cox proportional hazards regression analysis for Parkinson's disease risk in males with migraine

		Group	Participants (n)	PD diagnosis (n)	Person-years	Incidence rate per 1000 person-years	Model 3, HR (95% CI)	*P* value
**Age, year**	**<65**	Control	2 607 384	6791	23 928 876	0.284	1 (Ref.)	0.315
		Migraine	42 937	182	394 341	0.462	1.39(1.20–1.61)	
	**≥65**	Control	527 153	9024	4 308 337	2.095	1 (Ref.)	
		Migraine	16 423	389	131 064	2.968	1.27 (1.14–1.40)	
**Hypertension**	**No**	Control	1 998 720	7512	18 209 183	0.413	1 (Ref.)	0.201
		Migraine	32 063	208	289 345	0.719	1.22 (1.06–1.40)	
	**Yes**	Control	1 135 817	8303	10 028 030	0.828	1 (Ref.)	
		Migraine	27 297	363	236 060	1.538	1.36 (1.23–1.51)	
**Diabetes**	**No**	Control	2 700 080	12 489	24 455 425	0.511	1 (Ref.)	0.439
		Migraine	50 968	460	454 391	1.012	1.33 (1.21–1.46)	
	**Yes**	Control	434 457	3326	3 781 788	0.879	1 (Ref.)	
		Migraine	8392	111	71 014	1.563	1.22 (1.01–1.47)	
**Dyslipidemia**	**No**	Control	2 526 447	12 402	22 770 913	0.545	1 (Ref.)	0.012
		Migraine	44 960	397	398 245	0.997	1.22 (1.11–1.35)	
	**Yes**	Control	608 090	3413	5 466 299	0.624	1 (Ref.)	
		Migraine	14 400	174	127 160	1.368	1.54 (1.33–1.80)	
**BMI, kg/m^2**	**<25**	Control	1 961 304	10 283	17 564 396	0.585	1 (Ref.)	0.358
		Migraine	36 686	365	321 036	1.137	1.27 (1.14–1.41)	
	**≥25**	Control	1 173 233	5532	10 672 817	0.518	1 (Ref.)	
		Migraine	22 674	206	204 369	1.008	1.37 (1.20–1.58)	
**Current smoker**	**No**	Control	1 898 953	12 399	17 119 347	0.724	1 (Ref.)	0.203
		Migraine	40 548	464	358 963	1.293	1.27 (1.16–1.40)	
	**Yes**	Control	1 235 584	3416	11 117 866	0.307	1 (Ref.)	
		Migraine	18 812	107	166 443	0.643	1.46 (1.21–1.77)	

Abbreviations: Ref., reference; BMI, body mass index (calculated as weight in kilograms divided by height in meters squared); CI, confident interval; HR, hazard ratio; No., number; PD, Parkinson disease.
