# Supplementary material for: The association between migraine and Parkinson’s disease: a nationwide cohort study in Korea
Source: Epidemiol Health. 2023 Dec 18;46:e2024010. doi: 10.4178/epih.e2024010 (PMC10928470; doi:10.4178/epih.e2024010)
Supplement: Supplementary Material 5. — Multivariate Cox proportional hazards regression analysis for Parkinson’s disease risk in females with migraine [file epih-46-e2024010-Supplementary-5.pdf]

Supplementary Material 5. Multivariate Cox proportional hazards regression analysis for Parkinson's disease risk in females with migraine

		Group	Participants (n)	PD diagnosis (n)	Person-years	Incidence rate per 1000 person-years	Model 3, HR (95% CI)	*P* value
**Age, year**	**<65**	Control	2 214 690	5905	20 608 763	0.287	1 (Ref.)	0.038
		Migraine	115 403	499	1 075 722	0.464	1.47 (1.34–1.61)	
	**≥65**	Control	530 484	8944	4 644 560	1.926	1 (Ref.)	
		Migraine	39 430	903	343 960	2.625	1.30 (1.22–1.40)	
**Hypertension**	**No**	Control	1 873 297	6887	17 366 133	0.397	1 (Ref.)	0.174
		Migraine	90 920	523	842 829	0.621	1.30 (1.19–1.42)	
	**Yes**	Control	871 877	7 962	7 887 189	1.009	1 (Ref.)	
		Migraine	63 913	879	576 853	1.524	1.41 (1.31–1.51)	
**Diabetes**	**No**	Control	2 494 970	11 992	23 023 028	0.521	1 (Ref.)	0.852
		Migraine	139 247	1,129	1 282 041	0.881	1.37 (1.29–1.45)	
	**Yes**	Control	250 204	2857	2 230 294	1.281	1 (Ref.)	
		Migraine	15 586	273	137 641	1.983	1.35 (1.19–1.53)	
**Dyslipidemia**	**No**	Control	2 108 780	9598	19 429 939	0.494	1 (Ref.)	0.125
		Migraine	109 581	806	1 007 548	0.800	1.32 (1.23–1.41)	
	**Yes**	Control	636 394	5251	5 823 384	0.902	1 (Ref.)	
		Migraine	45 252	596	412 134	1.446	1.44 (1.32–1.56)	
**BMI, kg/m^2**	**<25**	Control	1 873 242	8973	17 229 716	0.521	1 (Ref.)	0.914
		Migraine	101 489	835	929 994	0.898	1.36 (1.27–1.46)	
	**≥25**	Control	871 932	5876	8 023 607	0.732	1 (Ref.)	
		Migraine	53 344	567	489 688	1.158	1.37 (1.26–1.49)	
**Current smoker**	**No**	Control	2 665 030	14 585	24 526 317	0.595	1 (Ref.)	0.426
		Migraine	150 495	1373	1 380 612	0.994	1.36 (1.29–1.44)	
	**Yes**	Control	80 144	264	727 006	0.363	1 (Ref.)	
		Migraine	4338	29	39 070	0.742	1.59 (1.09–2.34)	

Abbreviations: Ref., reference; BMI, body mass index (calculated as weight in kilograms divided by height in meters squared); CI, confident interval; HR, hazard ratio; No., number; PD, Parkinson disease
