# Supplementary material for: The association between migraine and Parkinson’s disease: a nationwide cohort study in Korea
Source: Epidemiol Health. 2023 Dec 18;46:e2024010. doi: 10.4178/epih.e2024010 (PMC10928470; doi:10.4178/epih.e2024010)
Supplement: Supplementary Material 6. — Methods, demographics, and results of the studies investigating Parkinson’s disease risk in patients with migraine [file epih-46-e2024010-Supplementary-6.pdf]

Supplementary Material 6. Methods, demographics, and results of the studies investigating Parkinson's disease risk in patients with migraine

First author (Year)	Design	Subjects (age)	Migraine No.	Control No.	Migraine diagnostic criteria	PD diagnostic criteria	Mean follow up period	Results: Migraine (total)	Results: Migraine with aura	Results: Migraine without aura
Scher (2014)	Prospective cohort study	Cohort in Reykjavik, Iceland (minimum age 43)	668	3,924	ICHD-1	Self-report	25 years	.	OR 2.53 (1.2–5.2)	OR 0.96 (0.2–4.1)
Wang (2016)	Retrospective cohort study (propensity score-matched controls)	Taiwanese residents (aged between 40 and 90 years)	41,292	41,019	ICD-9	ICD-9	2.6 years	HR 1.64 (1.25–2.14)	.	.
Current study	Retrospective cohort study	Korean residents (aged 40 years and over)	214,193	5,879,711	ICD-10	ICD-10, Rare incurable diseases system	9.1 years	HR 1.35 (1.29–1.41)	HR 1.51 (1.23–1.86)	HR 1.34 (1.28–1.40)

Abbreviations: PD, Parkinson's disease; ICHD-1, International Classification of Headache Disorders, first edition; ICD-9, International Classification of Diseases, ninth revision; ICD-10, International Classification of Diseases, tenth revision; HR, hazards ratio; OR, odds ratio
